# Research Progress of Bioactive Peptides in Improving Type II Diabetes

**DOI:** 10.3390/foods14030340

**Published:** 2025-01-21

**Authors:** Jiaxin Yu, Guoxing Chen, Yan Jin, Min Zhang, Tao Wu

**Affiliations:** 1 State Key Laboratory of Food Nutrition and Safety, Food Biotechnology Engineering Research Center of Ministry of Education, College of Food Science and Engineering, Tianjin University of Science & Technology, Tianjin 300457, China; 15602150842@163.com (J.Y.); cgx7896@163.com (G.C.); jinyan@tust.edu.cn (Y.J.); zm0102@tust.edu.cn (M.Z.); 2 School of Ocean and Environment, Tianjin University of Science & Technology, Tianjin 300457, China

**Keywords:** bioactive peptides, type II diabetes mellitus, insulin resistance, anti-inflammatory, glycolipid metabolism

## Abstract

Type II diabetes mellitus (T2DM) is a prevalent, long-standing metabolic condition marked by the body’s reduced response to insulin and inadequate insulin production, impacting a significant portion of the global population. Research has demonstrated that bioactive peptides play a crucial role in reducing blood sugar levels, enhancing insulin sensitivity, balancing lipid metabolism, and combating inflammation. These peptides also contribute to the enhancement of pancreatic islet function, lowering systemic inflammation by influencing various molecular signaling pathways. This paper provides an overview of recent advancements and potential applications of bioactive peptides in addressing T2DM. It highlights the diverse impacts of bioactive peptides sourced from different origins in combating diabetes. This comprehensive review offers theoretical substantiation and novel insights to support the future clinical utilization and exploration of bioactive peptides for T2DM management.

## 1. Introduction

Diabetes mellitus is a complex, long-term condition influenced by a combination of genetic and environmental elements. The global prevalence of diabetes has been on the rise in the 21st century, with an estimated 537 million individuals worldwide diagnosed with diabetes in 2021. This number is projected to surge by 46% to reach 783 million by 2045, and the onset of diabetes is occurring at progressively younger ages [1]. T2DM is a metabolic disorder distinguished by prolonged high blood sugar levels and elevated insulin levels stemming from compromised insulin function, secretion, or both factors [1]. The global incidence of T2DM is on the upswing across nations, largely attributed to sedentary habits and a widespread rise in obesity rates [2,3,4]. Currently, the primary treatment modalities for T2DM include insulin injections and oral anti-diabetic medications; however, many of these medications are associated with side effects, adverse reactions, and the development of drug resistance [5,6,7,8,9]. Noteworthy side effects of diabetes medications encompass heightened risk of hypoglycemia, skin rashes, drug-induced skin conditions, gastrointestinal disturbances, swelling, weight increase, and susceptibility to urinary tract infections among diabetic individuals [10,11,12,13,14]. Standard treatment approaches fail to address the personalized requirements of patients, leading to variations in the effectiveness of blood sugar regulation and an inability to prevent the progression of complications associated with the disease [11].

As research progresses, bioactive peptides have emerged as a focal point in enhancing T2DM management because of their natural, safe, and efficient attributes. Bioactive peptides from food are short amino acid sequences found in proteins that exhibit biological effects. Typically consisting of 2–20 amino acid residues, they are more readily digested and absorbed by the body compared to larger molecules [15]. Research indicates that incorporating grains, fruits, vegetables, fish, meat, eggs, milk, and other foods into the daily diet positively influences blood sugar control. Furthermore, bioactive peptides derived from natural sources, characterized by their small size, easy absorption, and adaptable structure, can effectively intervene and manage T2DM through various mechanisms [15,16,17,18,19,20,21,22]. Currently, the exploration of innovative food-derived bioactive peptides with enhanced biological potency, specificity, and bioavailability for preventing and treating metabolic conditions like T2DM is gaining momentum as a burgeoning research area.

Bioactive peptides sourced from food have the potential to enhance the management of T2DM through multiple mechanisms. Firstly, they can modulate energy metabolism and enhance glucose processing through uncoupling mechanisms. Specific bioactive peptides from food exhibit α-glucosidase inhibitory properties, slowing carbohydrate digestion and absorption to lower post-meal blood glucose levels [23]. Additionally, these peptides can boost glucose metabolism effectiveness, contributing to improved glucose processing efficiency. For instance, studies highlight that β-glucan-rich, low-starch bread enhances metabolic regulation and diminishes blood glucose levels in individuals with T2DM [24]. Conversely, bioactive peptides can enhance insulin sensitivity by influencing the PI3K/AKT and MAPK pathways [24]. Insulin plays a vital role in blood glucose regulation, and heightened insulin sensitivity facilitates improved cellular absorption and utilization of glucose, aiding in blood glucose management [25]. Furthermore, these peptides can be targeted to obstruct metabolic enzymes related to T2DM, including inhibiting α-glucosidase to delay carbohydrate absorption, reducing the rate of amylenzymatic hydrolysis to glucose, and impeding the escalation of blood glucose levels [26].

Currently, the prevalent techniques for creating bioactive peptides involve enzymatic and fermentation methods. By carefully selecting suitable proteases, refining the reaction parameters, and employing intricate enzymatic breakdown, highly potent small peptides can be synthesized [27]. Additionally, bioactive peptides find utility in crafting functional foods like whey protein and its associated bioactive peptides, known for their role in modulating blood glucose levels. These compounds exhibit diverse biological functions that can combat diet-induced obesity, improve glucose tolerance, and postpone the emergence of T2DM [28,29,30]. The advancement of functional foods enriched with bioactive peptides holds substantial importance in supporting the supplemental management of T2DM and elevating the nutritional worth of food products.

## 2. Overview of Bioactive Peptides

### 2.1. Characteristics and Sources of Bioactive Peptides

#### 2.1.1. Properties of Bioactive Peptides

Bioactive peptides are available from a variety of sources due to their low molecular weight and flexible structure compared to macromolecular proteins. Bioactive peptides have a high degree of structural diversity, which allows them to play specific roles against different biological targets (Table 1). For example, antimicrobial peptides (AMPs) can be extracted from a variety of sources, such as plants, animals, and micro-organisms, and have different structures and functions [31]. Pushpanathan et al. [31] also showed that certain AMPs can be used as antitumor, contraceptives, or drug delivery vehicles. This indicates that bioactive peptides not only have antibacterial activity but can also be used as signaling molecules, immunomodulators, growth factors, and other biological functions. Through 3D-QSAR analysis, specific physicochemical properties were found to be the key factors determining the antimicrobial activity and selectivity of AMPs [32]. This study demonstrates that bioactive peptides are often highly selective and specific for specific micro-organisms or cell types. This selectivity is primarily determined by the physicochemical interaction between the peptide and the cell membrane of interest. The molecular flexibility of bioactive peptides is critical to their biological activity. The flexibility of a molecule can enhance its ability to interact with the target molecule by altering its structural characteristics. Bioactive peptides of marine biological origin have antimicrobial activity and are able to inhibit microbial growth, which may help reduce the risk of infection in diabetes management [33].

#### 2.1.2. Sources of Bioactive Peptides

Bioactive peptides can be obtained from many different organisms, including animals, plants, micro-organisms, and marine organisms (Table 2). Bioactive peptides extracted from non-traditional food sources, such as those prepared from protein-rich non-traditional food sources such as *Bellamya bengalensis*, using enzymatic technology, have shown therapeutic functions, such as antihypertensive [39]. In addition, micro-organisms are also important sources of bioactive peptides, and many micro-organisms, such as bacteria, fungi, actinomycetes, and microalgae, are capable of synthesizing peptides with different biological activities [31]. It has been shown that marine plants, such as Sicilian marine fennel (*Crithmum maritimum*), have also been found to contain bioactive metabolites [39].

#### 2.1.3. Classification of Bioactive Peptides

BIOPEP-UWM currently lists 62 different feature categories, 4 of which are described below. These four categories were selected because they are important and representative in the field of biological functions. Antimicrobial peptides are related to biological defense and antimicrobial resistance. Plant growth promoting peptides help the green development of agriculture. Regulatory peptides regulate physiological activities in organisms. Growth factors promote biological growth, tissue repair, and medical regeneration.

##### Antimicrobial Peptides (AMPs)

Antimicrobial peptides are a class of short-chain polypeptides with broad-spectrum antimicrobial activity, which are widely found in nature, including plants, animals, and micro-organisms. These peptides inhibit or kill micro-organisms by interacting with their cell membranes and disrupting the integrity of their cell membranes [45]. Antimicrobial peptides are diverse in variety and structure, and their common feature is that they have cationic properties and are able to interact with negatively charged microbial cell membranes [41].

The mechanism of action of antimicrobial peptides is mainly to directly damage the cell membrane of micro-organisms and interfere with their internal structure. Some antimicrobial peptides are able to form transmembrane pores that cause cell contents to leak, thereby killing micro-organisms [46]. In addition, antimicrobial peptides can further inhibit microbial growth by binding to their DNA and interfering with their replication and transcription processes [43]. Antimicrobial peptides have also shown toxic effects on certain cancer cells, suggesting that they may also have potential applications in anticancer treatments.

##### Bioactive Peptides of Plant Origin

Plant-derived bioactive peptides have some potential to improve T2DM. Studies have shown [44] that natural peptides in legume seeds, such as aglycin, vglycin, and soymorphin-5, have been reported to have an effect on lowering blood glucose levels, improving insulin sensitivity, and glucose tolerance. These natural peptides promote β-cell proliferation and insulin secretion through their interaction with insulin and its receptors and therefore have a potential therapeutic effect on T2DM [44]. Studies have shown that plant-derived bioactive peptides have also been studied as potential drugs for the treatment of diabetes, playing a role in regulating immune responses, inhibiting tumor growth, and promoting apoptosis, showing promising applications in the treatment of diabetes [47].

##### Regulatory Peptides

Regulatory peptides are a class of small molecule peptides that have the function of regulating various physiological processes in living organisms. Regulatory peptides play an important role in improving T2DM through multiple pathways. On the one hand, they enhance insulin sensitivity and promote insulin secretion, thereby regulating blood glucose levels [48]. On the other hand, regulatory peptides can improve insulin resistance, reduce gluconeogenesis, and improve the body’s uptake and utilization of glucose. In addition, regulatory peptides can also regulate fat metabolism, reduce blood lipids, reduce fat accumulation, and alleviate the effects of obesity on diabetes [44]. At the same time, regulatory peptides also play an active role in regulating intestinal hormones and protecting pancreatic islet cells, improving the body’s metabolic function in an all-round way and providing new ideas and methods for the control and treatment of T2DM [49].

##### Growth Factors

Growth factors are a class of polypeptides with a variety of physiological functions that were originally described for their activity to promote cell division. These peptides not only play a role in cell division but are also involved in a variety of biological roles, such as regulating tissue morphology, differentiation, motility, and functional activity. Growth factors function through autocrine or paracrine mechanisms, and they function by binding to specific receptors on cell membranes, some of which also have kinase activity [50]. Together with hormones and neurotransmitters, growth factors play a fundamental role in cell-to-cell communication, with key functions including controlling the cell cycle, initiating mitosis, maintaining cell proliferation and survival, migration, differentiation, and apoptosis [51]. Growth factors play an important role in key events, such as follicular development, early embryogenesis, and implantation in reproductive activities, and although their mechanism of action and synergistic effects with other hormones are known, their full role has not been fully elucidated [52].

### 2.2. Common Bioactive Peptide Types

#### 2.2.1. Whey-Protein-Derived Bioactive Peptides

The bioactive peptides in whey protein directly promote insulin secretion and enhance insulin sensitivity. Melnik et al. [53] showed that branched-chain amino acids (BCAAs) in whey protein, such as leucine, isoleucine, and valine, can activate the mTORC1 signaling pathway in β cells and promote β-cell proliferation, insulin synthesis, and secretion. In addition, whey protein increases the secretion of glucose-dependent insulin-stimulated polypeptides (GIPs), thereby reducing postprandial blood glucose levels [54].

Whey protein can also improve glycemic control by modulating other metabolic pathways, and certain amino acid combinations (e.g., leucine, isoleucine, and valine) can mimic the insulin-boosting effects of whey protein without relying on incretin stimulation [54]. This mechanism suggests that whey protein can regulate blood sugar through a rapid postprandial amino acid response rather than by stimulating incretin hormone. In addition, whey protein may also work by inhibiting metabolic enzymes associated with T2DM. For example, some studies have suggested that the bioactive peptides in whey protein may inhibit the activity of α-glucosidase, thereby delaying carbohydrate absorption in the small intestine, decreasing the rate of amylenzymatic hydrolysis to glucose, and slowing the rise in blood glucose [55].

#### 2.2.2. C-Peptide

C-peptide is a product of proinsulin cleavage. Proinsulin is a single-chain polypeptide synthesized by pancreatic islet β cells, which is broken down into insulin and C-peptide, which is composed of 31 amino acids, by the action of enzymes. It is structurally stable; its metabolic processes in the body differ from insulin, mainly in the kidneys; and its metabolic clearance in the liver is lower than that of insulin. In recent years, it has been found that C-peptide has hormonal activity and plays a role in improving endothelial cell function and reducing peripheral blood flow, which has a positive effect on neuropathy and vascular complications in patients with type II diabetes mellitus [54]. It can improve endothelial cell function and vasodilation by increasing endothelial nitric oxide synthase activity, and it can also modulate inflammatory pathways to exert anti-inflammatory effects and protect nerves and blood vessels [56]. In diabetic neuropathy, C-peptide can improve nerve microcirculation, prevent axonal swelling, enhance nerve growth factor gene expression, and improve erythrocyte deformability, increasing tissue oxygen supply and uptake [55]. However, due to the presence of insulin resistance in T2DM, the role of C-peptide in T2DM may be limited, and its role in the development of atherosclerosis may have a potential pathogenic mechanism, and the mechanism of action is complex and two-sided [56].

#### 2.2.3. Yeast-Derived Bioactive Peptides

Yeast-derived bioactive peptides have a variety of biological activities, including antioxidant, antihypertensive, antimicrobial, and other properties. These peptides are mainly produced through the yeast fermentation process, which uses yeast extracts as raw materials to break down proteins through the action of microbial proteases to produce peptides with specific biological activities [57]. Yeast-derived bioactive peptides exhibit significant antioxidant effects. For example, antioxidant peptides produced during yeast fermentation are able to scavenge free radicals and inhibit lipid peroxidation, thereby reducing oxidative stress [58]. In addition, yeast-derived peptides also have hypotensive activity, which may be related to their inhibitory effect on angiotensin-converting enzyme (ACE) [57]. In terms of antimicrobials, yeast-derived bioactive peptides have also shown some potential. Studies have shown that certain yeast fermentation products are able to inhibit the growth of bacteria and fungi, which may be related to the structural and functional properties of peptides. In addition, the use of yeast extracts in food science has also highlighted their potential to enhance the nutritional value of foods and provide health benefits [59].

## 3. Effect of Bioactive Peptides on T2DM

### 3.1. Ameliorating Effect of Bioactive Peptides on T2DM

Bioactive peptides are a class of biomolecules with physiologically active short-chain amino acid sequences that have a positive effect on host health. Many studies in recent years have shown that bioactive peptides have significant potential in improving T2DM. The results of multiple animal experiments and clinical trials have shown that bioactive peptides can help control T2DM by regulating blood sugar, improving insulin sensitivity, and reducing inflammation (Table 3). Liao et al. [60] studied the establishment of a mouse model of T2DM by injection of streptozotocin (STZ), and the results showed that oral administration of pea peptides (1000 mg/kg BW) reduced blood glucose from 19.92 mmol/L to 14.3 mmol/L and significantly inhibited the phosphorylation expression of cAMP response element-binding protein(CREB) and glucose-6-phosphatase catalytic subunit (G6PC) in the hepatic gluconeogenesis signaling pathway. In addition, PPH also improves insulin sensitivity in T2DM mice by upregulating IRS-1, p-Akt, and phosphorylated forkhead box protein 1 levels. Other studies have shown that the small molecule insulin mimetic (Compound-2) shows significant improvement in a mouse model of non-hereditary T2DM, including reducing fasting and postprandial hyperglycemia, accelerating glucose clearance, and preventing weight gain and hypertrophy of adipose tissue, as well as the development of hepatic steatosis, compared to non-thiazolidinedione (nTZD) peroxisome proliferator-activated receptor-γ agonists. This suggests that bioactive peptides or similar compounds may improve the symptoms of T2DM by enhancing key steps in the insulin signaling pathway [61].

### 3.2. Bioactive Peptides Regulate the Pathway of Action in T2DM

**Figure 1:**
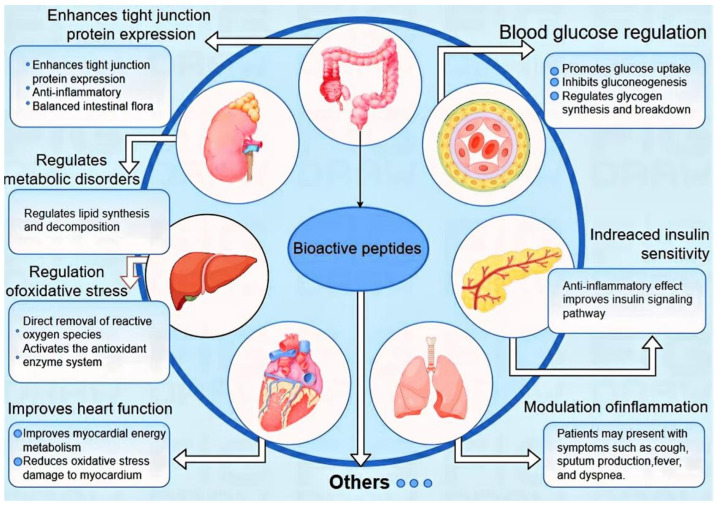
Regulatory mechanism of bioactive peptides in T2DM.

A large number of studies [64,65,66,67] have shown that bioactive peptides have the potential to improve T2DM and that they can achieve metabolic regulation in vivo through multiple pathways, as shown in Figure 1. Due to the complex pathogenesis of T2DM, the specific regulatory pathways of bioactive peptides still need to be further explored. Bioactive peptides function through multiple mechanisms, including promoting insulin secretion, improving insulin sensitivity, antioxidant and anti-inflammatory, regulating the intestinal microbiota, reducing blood lipid levels and improving fat distribution, and inhibiting intestinal glucose absorption [68,69,70]. These combined effects help regulate blood glucose and lipid metabolism, making bioactive peptides a potential multi-target, low-toxicity treatment option.

#### 3.2.1. Bioactive Peptides That Regulate Inflammation in T2DM

The antioxidant and anti-inflammatory properties of bioactive peptides are essential for T2DM management. Bioactive peptides in legumes and soy foods can improve glucose homeostasis and reduce inflammation by modulating the IR/IRS1 pathway, activating adiponectin and PPARα systems [44], and regulating blood glucose levels by inhibiting α-glucosidase activity or improving insulin resistance [71]. The marine-derived bioactive peptide has DPP4 inhibition, reduces inflammation, and exerts anti-inflammatory effects by reducing ROS production and inducing anti-inflammatory cytokine expression [72]. In addition, bioactive peptides, such as liraglutide, can reduce inflammation by modulating immune responses, such as modulating NADPH oxidase activity and altering macrophage polarization, which not only directly affects inflammatory signaling pathways but also indirectly acts by regulating immune cell function [73].

#### 3.2.2. Regulation of the Midgut Mucosal Barrier by Bioactive Peptides in Patients with T2DM

Many clinical studies have shown that the number of human gut microbiota is more than 10 times that of human cells, and these microbiota play an important role in metabolism and immune regulation [74,75,76,77]. Patients with T2DM often have impaired intestinal mucosal barrier function, resulting in dysbiosis of the intestinal flora. The disruption of abnormal gut metabolites and the intestinal barrier caused by this dysregulation further facilitates the entry of gut bacteria and their harmful metabolites into the circulatory system, which affects insulin sensitivity, glucose metabolism, and immune homeostasis, thereby causing damage to multiple organs [78]. In addition, numerous studies have shown that leguminous peptides are able to reduce the inflammatory response by modulating key pathways, such as NF-κB and MAPK, which play an important role in controlling inflammation-related cytokine production and enzyme activity [74,75]. By targeting these pathways, legume peptides can reduce the levels of pro-inflammatory markers, such as TNF-α and IL-6, thereby enhancing intestinal barrier function and reducing the risk of harmful substances entering the bloodstream.

#### 3.2.3. Effect of Bioactive Peptides on the Improvement of Metabolic Disorders in Patients with T2DM

Bioactive peptides have a potential positive effect on improving metabolic disorders in patients with type II diabetes mellitus (T2DM). These peptides can modulate and ameliorate metabolic abnormalities associated with T2DM through a variety of mechanisms. Bioactive peptides are able to reduce blood glucose levels by inhibiting the activity of enzymes such as DPP-IV, α-amylase, and α-glucosidase [79]. These enzymes play a key role in carbohydrate metabolism, and inhibiting them can reduce the rise in postprandial blood glucose, thereby contributing to the maintenance of a normal glycemic range [44]. Studies have shown that bioactive peptides can improve insulin secretion by regulating the function of pancreatic islet β cells. For example, bioactive peptides extracted from legumes, such as vglycin, are able to promote the proliferation of β cells and protect β-cell function by activating the IR/Akt/Erk signaling pathway [80]. In addition, dairy-derived bioactive peptides have also been shown to increase insulin secretion, suggesting that they may have potential benefits for improving islet β-cell function. In addition, bioactive peptides may also work by improving insulin sensitivity. For example, bioactive peptides can increase the concentration of glucagon-like peptide-1 (GLP-1) by inhibiting the DPP-4 enzyme, thereby enhancing insulin secretion and improving islet β-cell function [81].

#### 3.2.4. Regulation of Oxidative Stress by Bioactive Peptides in Patients with T2DM

Oxidative stress is one of the main variables in the development of diabetes. The activity of oxygen radicals causes lipid peroxidation, which leads to glycosylation of proteins, and alters the structure and function of membranes such as collagen and basement membranes, all of which contribute to the long-term difficulty of diabetes mellitus [81]. Bioactive peptides have the ability to inhibit reactive oxygen species (ROS), which is essential for reducing oxidative stress in diabetic patients. For example, three novel antioxidant peptides (YLVN, EEHLCFR, and TFY) extracted from pea protein hydrolysate exhibited significant cytoprotective effects in in vitro experiments. These peptides can significantly improve the survival rate of H_2_O_2_-induced oxidative damage in LO2 cells and reduce intracellular reactive oxygen species (ROS) levels, thereby attenuating oxidative damage [82].

#### 3.2.5. Effect of Bioactive Peptides on Blood Glucose Regulation in Patients with T2DM

Studies have shown that bioactive peptides function through a variety of mechanisms, including inhibition of α-glucosidase, inhibition of DPP-4 enzyme activity, and improvement of islet β-cell function [44,83]. For example, certain bioactive peptides are able to promote glucose uptake and utilization, reduce glucose production in the liver, and enhance insulin sensitivity by activating insulin signaling pathways. In addition, some plant-derived bioactive peptides have also been shown to mimic insulin-like proteins, which help maintain blood sugar levels [84].

#### 3.2.6. Effect of Bioactive Peptides on Enhanced Insulin Sensitivity in Patients with T2DM

##### Improved Insulin Signaling

**Figure 2:**
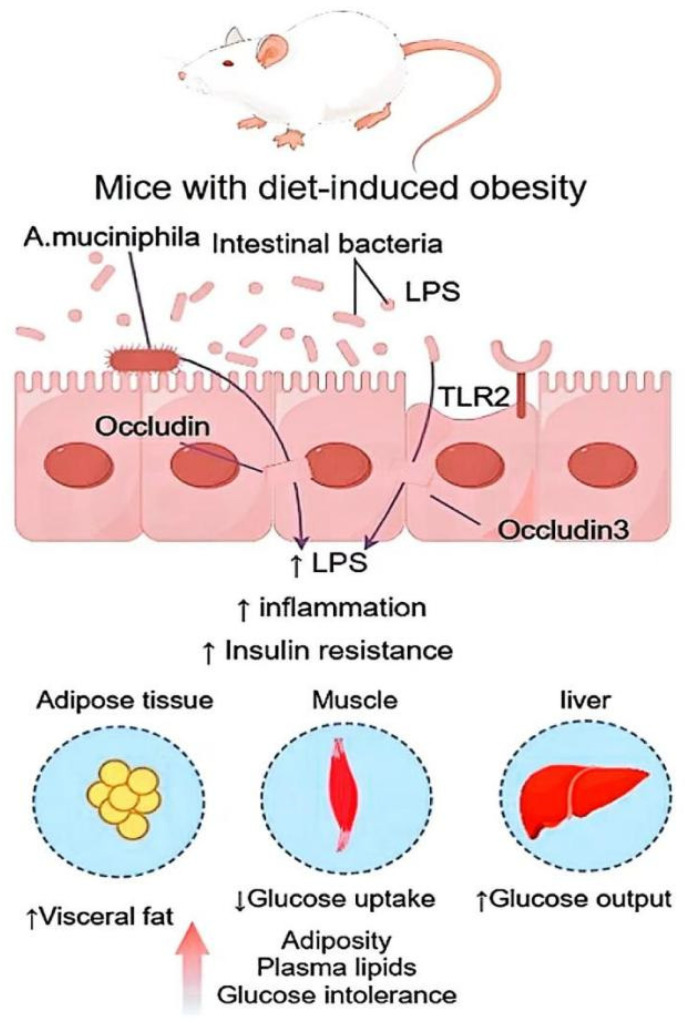
Effect of bioactive peptides on insulin resistance in patients with T2DM.

Barbuio et al. [83] demonstrated that, after a 10-day treatment with infliximab, the expression levels of pro-inflammatory markers, including TNF-α, IL-6, and IL-1β, were significantly reduced in the livers of rats subjected to a high-fat diet. This modulation was accompanied by a reduction in hepatic fibrosis and fat accumulation. The JAK2/STAT3 and insulin receptor (IR)/IR substrate/Akt/FOXO1 pathways also enhance insulin signaling. When insulin binds to insulin receptors on cell membranes, it activates a series of downstream signaling pathways, such as the PI3K/Akt pathway and the MAPK pathway, to regulate processes such as glucose metabolism, lipid metabolism, and protein synthesis [85]. Dysfunction of these signaling pathways frequently leads to insulin resistance, which limits the physiological effects of insulin and impairs blood glucose control. Through methods, bioactive peptides promote insulin signaling, which in turn increases insulin action and insulin sensitivity [86]. For example, dietary bioactive peptides can enhance β-cell function, increase insulin secretion, and improve the effects of insulin [87]. Some food-derived bioactive peptides, like those found in whey protein, fish, and sea cucumber, can stimulate the PI3K/Akt signaling pathway and enhance the effects of insulin [88]. Bioactive peptides lower blood glucose levels by improving insulin receptor function, promoting glucose absorption and utilization, and increasing the activity of this signaling system. Whey protein peptides, for instance, contain certain amino acids that can encourage the phosphorylation of insulin receptors. This subsequently activates the PI3K/Akt pathway, increases insulin signaling, and raises insulin sensitivity [89]. By enhancing the effects of insulin and preventing the inflammatory response in adipose tissue, bioactive peptides may also help lower blood sugar levels. By attaching to insulin receptors, whey protein peptides improve insulin signaling and prevent the development of insulin resistance, offering a novel approach to the management of T2DM (Figure 2).

##### Regulation of Metabolic Pathways

In T2DM, there is a dysregulation of multiple metabolic processes. Bioactive peptides can intervene at various critical points. Firstly, they play a crucial role in regulating cellular redox homeostasis, which is often disrupted in T2DM due to chronic hyperglycemia-induced oxidative stress [44]. By acting as antioxidants or interacting with redox-regulating enzymes like glutathione peroxidase and superoxide dismutase, bioactive peptides can scavenge reactive oxygen species (ROS), protecting cells from oxidative damage [90]. This, in turn, ensures the proper functioning of enzymes and substrates involved in glycolysis, the citric acid cycle, and fatty acid metabolism, allowing these processes to occur more efficiently. Secondly, bioactive peptides can enhance insulin sensitivity. They may do so by interacting with insulin receptors or intracellular signaling pathways, facilitating glucose uptake by cells such as adipocytes and muscle cells. Additionally, some bioactive peptides can regulate hepatic glucose production, inhibiting excessive glycogenolysis and gluconeogenesis in the liver [90].

##### Antioxidant Effect

Bioactive peptides have certain antioxidant activity and can improve the effect of insulin by scavenging free radicals and inhibiting oxidative stress [91]. For example, some peptides derived from soybeans, dairy products, and marine organisms have strong antioxidant capacity. These bioactive peptides can help improve insulin sensitivity by inhibiting lipid peroxidation, decreasing ROS levels, and activating the antioxidant enzyme system, thereby reducing the negative effects of oxidative stress on the insulin signaling pathway [87]. In addition, bioactive peptides such as peptides of hazelnut powder hydrolysate [92] can also enhance the antioxidant capacity of cells and reduce the accumulation of free radicals by regulating intracellular antioxidant enzyme systems, such as superoxide dismutase (SOD), catalase (CAT), and glutathione peroxidase (GPx) [87]. Through these effects, bioactive peptides not only help reduce insulin resistance but also improve diabetes-related chronic complications.

### 3.3. Novel Anti-T2DM Drugs Based on Bioactive Peptides That Are Under Development or Have Been Marketed

#### 3.3.1. Glucagon-Like Peptide-1 Receptor Agonist (GLP-1 RA)

GLP-1 RA lowers blood glucose levels by activating glucagon-like peptide-1 receptor (GLP-1R), enhancing insulin secretion and suppressing glucagon release [83]. Studies have shown that GLP-1 RA is able to reduce inflammatory markers and oxidative stress levels in diabetes models [93]. In addition, GLP-1 RA was able to improve the blood lipid profile of patients, including decreasing low-density lipoprotein (LDL) and increasing high-density lipoprotein (HDL) levels, which are important indicators of cardiovascular disease risk [93]. Multiple clinical trials have demonstrated that GLP-1 RA can significantly reduce the incidence of major adverse cardiovascular events (MACEs) in patients with T2DM, including cardiovascular death, nonfatal myocardial infarction, and nonfatal stroke [94].

#### 3.3.2. Sodium-Glucose Cotransporter 2 Inhibitor (SGLT-2i)

SGLT-2i reduces glucose reabsorption by inhibiting sodium-glucose cotransporter 2 in the proximal tubule of the kidney, thereby increasing urinary glucose excretion and lowering blood glucose levels [94]. This action is independent of the action of insulin; therefore, it is effective even in the presence of impaired β-cell function. In addition, SGLT-2i can reduce the risk of insulin-induced weight gain and fluid retention, as well as the risk of hypoglycemia [95]. SGLT-2i also improves the function of pancreatic islet β cells and reduces the stress on β cells [96]. This may help slow the progression of diabetes and improve blood sugar control. SGLT-2i also provides additional metabolic benefits by reducing blood pressure, improving lipid profiles, and reducing arterial stiffness and endothelial dysfunction [97].

### 3.4. Prospects for the Application of Bioactive Peptides in T2DM

Bioactive peptides have significant application potential in the prevention and treatment of T2DM mellitus and have become one of the hot spots in the current research on metabolic diseases. The pathogenesis of T2DM mellitus is complex, involving insulin resistance, impaired pancreatic β-cell function, chronic inflammation, and oxidative stress, and bioactive peptides can intervene in these key pathological links through multiple pathways and multi-target mechanisms [97]. The challenge for the food industry today is how to incorporate these bioactive peptides without compromising the organoleptic properties, convenience, bioavailability, and safety of food. Opportunities and challenges are always interdependent, and there are sufficient available data to show that bioactive peptides of various natural origins have a range of biological and functional properties, which undoubtedly underscores their huge scope of applications in the food and pharmaceutical industries in the future [98]. If the scientific understanding and clinical effectiveness of these peptides can be further improved, they are expected to be used as a unique functional peptide nutrition strategy for the treatment of T2DM and other metabolic disorders. For example, the α-glucosidase inhibitory peptide in black bean protein hydrolysate has shown the ability to lower blood sugar and promote glucose uptake [44].

#### 3.4.1. As a Functional Food and Nutritional Supplement

Functional foods and bioactive compounds have been shown to be adjunctive treatments for T2DM due to their biological properties. These foods and nutritional supplements are able to prevent the development of long-term diabetic complications, including cardiovascular disease, neuropathy, nephropathy, and retinopathy, by improving postprandial hyperglycemia, regulating carbohydrate and lipid metabolism, improving insulin sensitivity, and attenuating oxidative stress and inflammatory processes [99]. In particular, legume seeds (peas, beans, lentils, peanuts), as unique functional foods rich in bioactive proteins and peptides, functional fibers, non-digestible carbohydrates, and phytochemicals, have been shown to reduce postprandial blood glucose and insulin levels, increase insulin sensitivity, regulate lipid and lipoprotein metabolism, and reduce oxidative stress [100,101]. Bioactive peptides extracted from distilled grains show good α-glucosidase inhibition and antioxidant properties, enabling the improvement of insulin resistance and thus enhancing the management of type II diabetes [94]. In addition, food-derived bioactive peptides have additional beneficial effects on the management of T2DM due to their ability to control multiple mechanisms involved in carbohydrate metabolism [101]. Therefore, the inclusion of these functional foods and nutritional supplements in the dietary treatment plan of diabetic patients in the future is a comprehensive and innovative management strategy [99,100].

#### 3.4.2. As a New Direction for Drug Development

Insulin-like growth factors (IGFs) and their binding proteins (IGFBPs) play an important role in the pathogenesis of T2DM, and these factors and proteins influence the development of diabetes mellitus by regulating the insulin signaling pathway [102]. For example, IGF-I has been reported to improve insulin sensitivity in healthy subjects and patients with T2DM [101]. IGFBPs have been recognized as therapeutic targets in metabolic syndrome and T2DM, and they can be used as part of combination therapies with other anti-diabetic drugs [102]. On the other hand, new anti-diabetic drugs, such as sodium-glucose cotransporter 2 inhibitors (SGLT2i) and glucagon-like peptide-1 receptor agonist (GLP1RA), have shown significant efficacy in clinical practice, and these drugs not only improve glycemic control but also benefit cardiovascular and renal function [103]. This suggests that bioactive peptides and their derivatives have the potential to improve the overall health of patients with T2DM. Researchers are also exploring new ways to treat T2DM by improving β-cell function. For example, G(q)-coupled receptors designed for chronic activation can significantly improve β-cell function and glucose homeostasis [104]. This strategy provides a theoretical basis for the development of new anti-diabetic drugs that target specific cellular pathways. Therefore, bioactive peptides can be used not only as a single treatment but also in combination with other drugs to improve the therapeutic effect and reduce side effects. Future research needs to further explore the specific mechanism of action and optimal application of these bioactive molecules to achieve more effective diabetes management [102].

### 3.5. Clinical Application of Drugs Based on Peptides in the Intervention of T2DM

Liraglutide is a human glucagon-like peptide-1 (GLP-1) analog with a highly homologous amino acid sequence to human endogenous GLP-1 and exerts corresponding physiological functions by binding to the GLP-1 receptor. Liraglutide and exenatide, as GLP-1 receptor agonists, have shown significant efficacy and safety in the clinical application of T2DM and have good development prospects. Liraglutide is effective in reducing postprandial and fasting blood glucose levels by prolonging gastric emptying time, increasing insulin secretion, and improving islet β-cell function [105]. Its once-daily dosing improved patient compliance and was well tolerated, with no hypoglycemic events reported [106]. In phase III clinical trials, liraglutide showed significant reductions in glycosylated hemoglobin (HbA1c) and body weight [107]. Liraglutide is more effective than rosiglitazone alone or placebo when used in combination with sulfonylureas [108]. In addition, Candeias et al. [109] showed that liraglutide was superior to exenatide in improving glycemic control in patients with T2DM. Liraglutide has been approved for use in adolescent patients with T2DM and has shown good glycemic control with minor side effects [106,110]. This opens up new treatment options for adolescent patients, especially when traditional medications do not achieve optimal results. Despite its significant efficacy, liraglutide may cause kidney injury and acute kidney injury and requires monitoring and management in clinical practice [109].

## 4. Conclusions

Bioactive peptides, as a novel approach to treating T2DM, have garnered significant attention in the realm of diabetes care due to their innate safety, versatility, and therapeutic potential. The above article demonstrates the ability to not only efficiently enhance blood glucose control but also holistically modulate the body’s metabolic functions through diverse pathways. This includes boosting insulin sensitivity, curbing chronic inflammation, optimizing lipid metabolism, and fortifying intestinal health.

Recent studies have validated the beneficial impacts of diverse bioactive peptides sourced from nature in reducing blood sugar levels, enhancing pancreatic islet function, and mitigating complications associated with diabetes. For instance, specific peptides derived from dietary proteins can mitigate the postprandial rise in blood glucose levels by inhibiting glycosidase activity. Other peptides stimulate insulin release indirectly through the activation of GLP-1 receptors. Furthermore, active peptides sourced from marine sources exhibit promise in modulating lipid metabolism and ameliorating non-alcoholic fatty liver disease. This presents novel opportunities for the holistic management of intricate metabolic conditions. Despite significant advancements, numerous challenges persist in this field. The stability, availability in the body, and metabolic routes of bioactive peptides in humans are yet to be comprehensively elucidated. Moreover, the integration of bioengineering and nanotechnology into the development of peptide-based therapeutics holds the potential to improve their stability, enable precise targeting, reduce adverse effects, and enhance therapeutic efficacy. These advancements highlight an expanding role for bioactive peptides in the management of diabetes and its associated complications, offering promising prospects for individuals living with the condition worldwide.

While current studies support its therapeutic potential, larger, longer-term clinical trials are needed to further validate its efficacy and safety. It is of great significance to carry out clinical trials to verify the therapeutic effect of bioactive peptides, provide a basis for clinical application, and promote scientific progress. In the future, it is hoped that relevant research institutions, enterprises, and government departments will increase investment in bioactive peptide research and actively carry out clinical trials to provide new ways and methods for the treatment of T2DM.

## Figures and Tables

**Table 1:** Functions of bioactive peptides.

Functional Categories	Description of the Function	References
Antimicrobial peptides	It has a broad-spectrum antibacterial effect, such as anti-Escherichia coli and Staphylococcus aureus.	[34]
Antioxidant peptides	It has antioxidant function, can scavenge free radicals and reduce oxidative stress.	[35]
Signal peptides	Stimulates collagen and elastin synthesis, promotes keratinocyte and epidermal cell proliferation, and regulates melanin production.	[36]
Carrier peptides	Delivers trace elements to promote wound healing and enzymatic processes, improving skin elasticity.	[36]
Neurotransmitter inhibitory peptides	Provides a moisturizing effect, stimulates collagen and elastin synthesis, and inhibits melanin synthesis.	[36]
Enzyme inhibitory peptides	Reduces collagen and elastin breakdown to provide hydration.	[36]
Antihypertensive peptides	Has antihypertensive functions, such as antihypertensive peptides identified from soybean protein hydrolysate.	[37]
Anti-hyperlipidemic peptides	It has the function of lowering cholesterol and hyperlipidemia, such as plant-derived anti-hyperlipidemic peptides.	[34]
Processed peptides for dairy products	Activated by proteases in dairy processing to improve the taste, texture, and microbial community of dairy products.	[38]

**Table 2:** Comparison of common preparation methods for bioactive peptides.

Methods	Merit	Shortcoming	Application	References
Chemical hydrolysis	Low cost and simple operation	The hydrolysis conditions are difficult to control; amino acids are easy to destroy; and the product quality is unstable, which is not suitable for industrial production	It is mainly used for laboratory research	[40]
Enzymatic digestion	The conditions are mild; the reaction time is short; there is no racemic effect, no destruction of amino acids; the product is of high purity, easy to separate; and there is no pollution. It is highly safe, easily digested and absorbed by the human body, and the production conditions are mild and easy to control	It is necessary to study the effects of enzyme types, dosages, substrate concentrations, temperatures, pH, time, and other parameters on the preparation of peptides, and the quality and purity of peptides prepared under different conditions are different	The most commonly used preparation method, suitable for a variety of raw materials, such as millet protein, egg white protein, soy protein, etc.	[41]
Microbial fermentation	The cost of raw materials is low; the process is simple; the conditions are mild; and the protease yield is high. The mixed enzymes produced by microbial metabolic activities can release bioactive peptides in aqueous conditions, and at the same time, micro-organisms can improve their growth and enzyme production capabilities with the help of peptide hydrolysate, and they cooperate with each other for higher efficiency	The microbial fermentation and metabolic process is complex; the products are difficult to control; and many impurities will be generated, which is difficult for the later separation and purification	It is suitable for the preparation of soybean protein, konjac flour, sweet potato protein, and other raw materials	[42]
Chemical synthesis via solid-phase method	High purity (>98%), suitable for large-scale production	The cost is high, and the time is long	Preparation of bioactive peptides, such as XOI inhibitory peptides	[43]
Organic synthesis	Peptides with specific structures can be designed with high flexibility	Costly, time-consuming, and unsustainable	Preparation of marine bioactive peptides	[44]

**Table 3:** Effects of different bioactive peptides on T2DM.

Subjects	Name of Bioactive Peptides	Research Results	References
STZ/HFD-induced diabetic BALB/c mice (50 mg/kg/day, 4 weeks)	Soy peptides	BG↓ p-Akt, OGTT, insulin tolerance, p-IR↑ p-IRS1, GLUT4, glucose uptake↑	[61]
Mice	Donkey milk protein peptides	Blood sugar levels↓ insulin sensitivity, insulin resistance↑	[61]
High-fat diet and streptozotocin (dose 1000 mg/kg BW) were induced in mice	Pea protein peptides	Insulin sensitivity in T2DM mice↓ food intake and blood glucose↓	[57]
A rat model of obese type II diabetes	Whey protein bioactive peptides	Fasting blood glucose, glycosylated hemoglobin↓insulin secretion, insulin resistance↑diversity of intestinal flora and number of beneficial bacteria↑	[62]
Streptozotocin (STZ)-induced diabetic rats	Bitter melon polypeptide	Body weight↓ oral carbohydrate tolerance↑ blood sugar levels, glycosylated hemoglobin, lactate dehydrogenase↓ hemoglobin levels, glycogen and hexokinase and glycogen synthase activity↑	[63,64]
A high-fat, high-fructose diet induced in rats	Goby muscle protein hydrolysate	α-Amylase levels, blood glucose, liver glycogen content↓	[64]

↑: facilitation, ↓: inhibition.

## Data Availability

The original contributions presented in this study are included in the article. Further inquiries can be directed to the corresponding author.
